# Supplementary material for: Four-Dimensional Superimposition Techniques to Compose Dental Dynamic Virtual Patients: A Systematic Review
Source: J Funct Biomater. 2023 Jan 6;14(1):33. doi: 10.3390/jfb14010033 (PMC9861356; doi:10.3390/jfb14010033)
Supplement: Supplementary file 1 [file jfb-14-00033-s001.zip › Tables S2 and S3 revised.pdf]

Table S2. Joanna Briggs Institute Critical Appraisal Checklist for Quasi-Experimental Studies (non-randomized experimental studies)

Question	Answer (YesⓎ, NoⓃ, UnclearⓊ, or not applicableⓍ)
Q1. Is it clear in the study what is the "cause" and what is the 'effect' (i.e., there is no confusion about which variable comes first)?	
Q2. Were the participants included in any similar comparisons?	
Q3. Were the participants included in any comparisons receiving similar treatment/care other than the exposure or intervention of interest?+	
Q4. Was there a control group?	
Q5. Were there multiple measurements of the outcome both before and after the intervention/exposure?	
Q6. Was follow up complete and if not, were differences between groups in terms of their follow up adequately described and analyzed?	
Q7. Were the outcomes of participants included in any comparisons measured in the same way?	
Q8. Were outcomes measured in a reliable way?	
Q9. Was appropriate statistical analysis used?	

Non-randomized experimental studies	Q1	Q2	Q3 +	Q4	Q5	Q6	Q7	Q8	Q9	Percentage of yes	Risk of Bias*
Li J[19] /2022	Ⓨ	Ⓨ	Ⓝ	Ⓨ	Ⓧ	Ⓧ	Ⓨ	Ⓨ	Ⓨ	77.8%	moderate
Kim SH[25] /2020	Ⓨ	Ⓨ	Ⓝ	Ⓨ	Ⓧ	Ⓧ	Ⓨ	Ⓨ	Ⓨ	77.8%	moderate
Lee SJ[26] /2019	Ⓨ	Ⓨ	Ⓝ	Ⓨ	Ⓧ	Ⓧ	Ⓨ	Ⓨ	Ⓨ	77.8%	moderate
Olszewski R[29] /2008	Ⓨ	Ⓤ	Ⓤ	Ⓨ	Ⓧ	Ⓧ	Ⓨ	Ⓨ	Ⓨ	55.6%	substantial
Fushima K[18] /2007	Ⓨ	Ⓤ	Ⓝ	Ⓨ	Ⓧ	Ⓧ	Ⓨ	Ⓨ	Ⓨ	66.7%	moderate
Lam WYH[32] /2016	Ⓨ	Ⓨ	Ⓝ	Ⓨ	Ⓧ	Ⓧ	Ⓨ	Ⓨ	Ⓤ	66.7%	moderate
Lam WYH[33] /2018	Ⓨ	Ⓨ	Ⓝ	Ⓨ	Ⓧ	Ⓧ	Ⓨ	Ⓨ	Ⓤ	66.7%	moderate
He S[35] /2016	Ⓨ	Ⓨ	Ⓝ	Ⓨ	Ⓧ	Ⓧ	Ⓨ	Ⓨ	Ⓤ	66.7%	moderate
Amezua X[37] /2021	Ⓨ	Ⓨ	Ⓝ	Ⓨ	Ⓧ	Ⓧ	Ⓨ	Ⓨ	Ⓨ	77.8%	moderate
Savoldelli C[39] /2012	Ⓨ	Ⓨ	Ⓝ	Ⓨ	Ⓧ	Ⓧ	Ⓨ	Ⓨ	Ⓤ	66.7%	moderate

+ It represents high risk when the answer to Q3 is yes. Therefore, the yes and no of Q3 are swapped when calculating percentage of yes.

* 0%-39% may represent a high risk of bias; 40%-59% may represent a substantial risk of bias; 60%-79% may represent a moderate risk of bias; 80%-100% may represent a low risk of bias.

Table S3. Joanna Briggs Institute Critical Appraisal Checklist for case reports

Question	Answer (YesⓎ, NoⓃ, UnclearⓊ, or not applicableⓍ)
Q1. Were patient's demographic characteristics clearly described?	
Q2. Was the patient's history clearly described and presented as a timeline?	
Q3. Was the current clinical condition of the patient on presentation clearly described?	
Q4. Were diagnostic tests or assessment methods and the results clearly described?	
Q5. Was the intervention(s) or treatment procedure(s) clearly described?	
Q6. Was the post-intervention clinical condition clearly described?	
Q7. Were adverse events (harms) or unanticipated events identified and described?	
Q8. Does the case report provide takeaway lessons?	

Case reports	Q1	Q2	Q3	Q4	Q5	Q6	Q7	Q8	Percentage of yes	Risk of Bias*
Zambrana N[20] /2022	Ⓝ	Ⓝ	Ⓝ	Ⓧ	Ⓨ	Ⓨ	Ⓝ	Ⓨ	37.5%	high
Kim JE[21] /2019	Ⓝ	Ⓝ	Ⓝ	Ⓧ	Ⓨ	Ⓨ	Ⓝ	Ⓨ	37.5%	high
Revilla-Leon M[22] /2022	Ⓝ	Ⓝ	Ⓝ	Ⓧ	Ⓨ	Ⓨ	Ⓝ	Ⓨ	37.5%	high
Revilla-Leon M[23] /2022	Ⓝ	Ⓝ	Ⓝ	Ⓧ	Ⓨ	Ⓨ	Ⓝ	Ⓨ	37.5%	high
Lepidi L[24] /2021	Ⓝ	Ⓝ	Ⓨ	Ⓧ	Ⓨ	Ⓨ	Ⓝ	Ⓨ	50%	substantial
Li J[27] /2020	Ⓨ	Ⓝ	Ⓨ	Ⓧ	Ⓨ	Ⓨ	Ⓝ	Ⓨ	62.5%	moderate
Li J[28] /2021	Ⓝ	Ⓝ	Ⓝ	Ⓧ	Ⓨ	Ⓨ	Ⓝ	Ⓨ	37.5%	high
Kois JC[30] /2022	Ⓝ	Ⓝ	Ⓝ	Ⓧ	Ⓨ	Ⓨ	Ⓝ	Ⓨ	37.5%	high
Kwon JH[31] /2019	Ⓝ	Ⓝ	Ⓝ	Ⓧ	Ⓨ	Ⓨ	Ⓝ	Ⓨ	37.5%	high
Shao J[34] /2019	Ⓨ	Ⓝ	Ⓨ	Ⓨ	Ⓨ	Ⓨ	Ⓝ	Ⓨ	75%	moderate
Park JH[36] /2021	Ⓨ	Ⓝ	Ⓨ	Ⓨ	Ⓨ	Ⓨ	Ⓝ	Ⓨ	75%	moderate
Dai F[38] /2016	Ⓨ	Ⓝ	Ⓨ	Ⓧ	Ⓨ	Ⓨ	Ⓝ	Ⓨ	62.5%	moderate
Terajima M[4] /2008	Ⓨ	Ⓨ	Ⓨ	Ⓨ	Ⓨ	Ⓨ	Ⓝ	Ⓨ	87.5%	low
Perez-Giugovaz MG[40]	Ⓝ	Ⓝ	Ⓝ	Ⓧ	Ⓨ	Ⓨ	Ⓝ	Ⓨ	37.5%	high

/2021										
Solaberrieta E[41] /2015	Ⓝ	Ⓝ	Ⓝ	Ⓧ	Ⓨ	Ⓨ	Ⓝ	Ⓨ	37.5%	high
Granata S[42] /2020	Ⓝ	Ⓝ	Ⓨ	Ⓨ	Ⓨ	Ⓨ	Ⓝ	Ⓨ	62.5%	moderate
Noguchi N[43] /2007	Ⓝ	Ⓝ	Ⓨ	Ⓧ	Ⓨ	Ⓨ	Ⓝ	Ⓨ	50%	substantial

* 0%-39% may represent a high risk of bias; 40%-59% may represent a substantial risk of bias; 60%-79% may represent a moderate risk of bias; 80%-100% may represent a low risk of bias.
