# Supplementary material for: Four-Dimensional Superimposition Techniques to Compose Dental Dynamic Virtual Patients: A Systematic Review
Source: J Funct Biomater. 2023 Jan 6;14(1):33. doi: 10.3390/jfb14010033 (PMC9861356; doi:10.3390/jfb14010033)
Supplement: Supplementary file 1 [file jfb-14-00033-s001.zip › Figure S1.Snowball procedure.pdf]

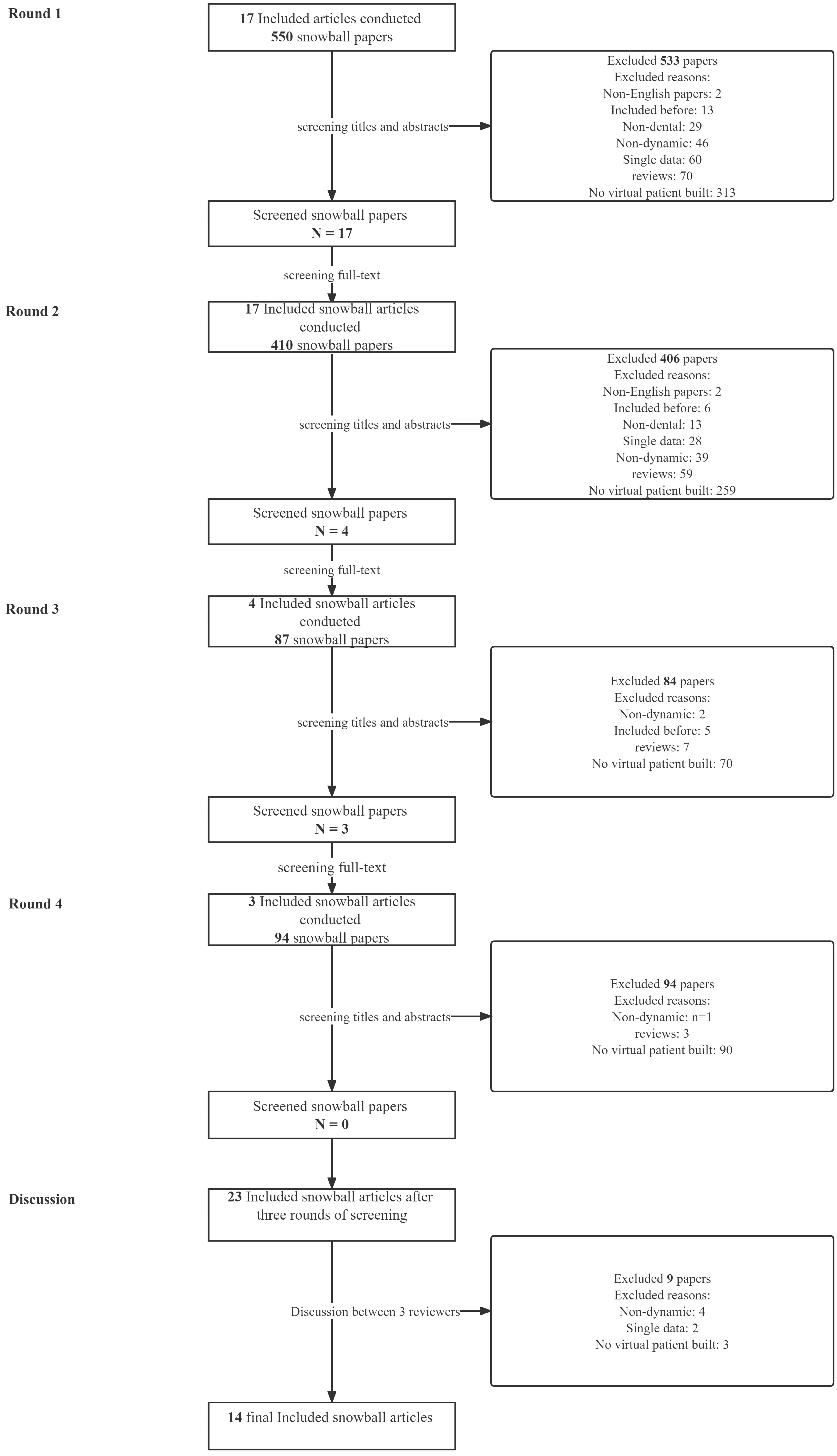
Round 1
17 Included articles conducted
550 snowball papers
screening titles and abstracts
Excluded 533 papers
Excluded reasons:
Non-English papers: 2
Included before: 13
Non-dental: 29
Non-dynamic: 46
Single data: 60
reviews: 70
No virtual patient built: 313
Screened snowball papers
N = 17
screening full-text
Round 2
17 Included snowball articles conducted
410 snowball papers
screening titles and abstracts
Excluded 406 papers
Excluded reasons:
Non-English papers: 2
Included before: 6
Non-dental: 13
Single data: 28
Non-dynamic: 39
reviews: 59
No virtual patient built: 259
Screened snowball papers
N = 4
screening full-text
Round 3
4 Included snowball articles conducted
87 snowball papers
screening titles and abstracts
Excluded 84 papers
Excluded reasons:
Non-dynamic: 2
Included before: 5
reviews: 7
No virtual patient built: 70
Screened snowball papers
N = 3
screening full-text
Round 4
3 Included snowball articles conducted
94 snowball papers
screening titles and abstracts
Excluded 94 papers
Excluded reasons:
Non-dynamic: n=1
reviews: 3
No virtual patient built: 90
Screened snowball papers
N = 0
Discussion
23 Included snowball articles after three rounds of screening
Discussion between 3 reviewers
Excluded 9 papers
Excluded reasons:
Non-dynamic: 4
Single data: 2
No virtual patient built: 3
14 final Included snowball articles
